# β_3_-Adrenergically induced glucose uptake in brown adipose tissue is independent of UCP1 presence or activity: Mediation through the mTOR pathway

**DOI:** 10.1016/j.molmet.2017.02.006

**Published:** 2017-03-30

**Authors:** Jessica M. Olsen, Robert I. Csikasz, Nodi Dehvari, Li Lu, Anna Sandström, Anette I. Öberg, Jan Nedergaard, Sharon Stone-Elander, Tore Bengtsson

**Affiliations:** 1 Department of Molecular Biosciences, The Wenner-Gren Institute, Stockholm University, SE-106 91, Stockholm, Sweden; 2 Karolinska Experimental Research and Imaging Center, Karolinska University Hospital, Solna, SE-171 76, Stockholm, Sweden; 3 Department of Clinical Neurosciences, Karolinska Institutet, SE-171 77, Stockholm, Sweden; 4 Department of Neuroradiology, Karolinska University Hospital Solna, SE-171 76, Stockholm, Sweden

**Keywords:** Brown adipose tissue, Uncoupling protein 1, Glucose uptake, Adrenergic signaling, Positron emission tomography

## Abstract

**Objective:**

Today, the presence and activity of brown adipose tissue (BAT) in adult humans is generally equated with the induced accumulation of [2-^18^F]2-fluoro-2-deoxy-d-glucose ([^18^F]FDG) in adipose tissues, as investigated by positron emission tomography (PET) scanning. In reality, PET-FDG is currently the only method available for *in vivo* quantification of BAT activity in adult humans. The underlying assumption is that the glucose uptake reflects the thermogenic activity of the tissue.

**Methods:**

To examine this basic assumption, we here followed [^18^F]FDG uptake by PET and by tissue [^3^H]-2-deoxy-d-glucose uptake in wildtype and UCP1(−/−) mice, i.e. in mice that do or do not possess the unique thermogenic and calorie-consuming ability of BAT.

**Results:**

Unexpectedly, we found that β_3_-adrenergically induced (by CL-316,243) glucose uptake was UCP1-independent. Thus, whereas PET-FDG scans adequately reflect glucose uptake, this acute glucose uptake is not secondary to thermogenesis but is governed by an independent cellular signalling, here demonstrated to be mediated via the previously described KU-0063794-sensitive mTOR pathway.

**Conclusions:**

Thus, PET-FDG scans do not exclusively reveal active BAT deposits but rather any tissue possessing an adrenergically-mediated glucose uptake pathway. In contrast, we found that the marked glucose uptake-ameliorating effect of prolonged β_3_-adrenergic treatment was UCP1 dependent. Thus, therapeutically, UCP1 activity is required for any anti-diabetic effect of BAT activation.

## Highlights

• β_3_-adrenergically glucose uptake in BAT is UCP1-independent.
• Glucose uptake is not secondary to thermogenesis but is through the mTOR pathway.
• Both glucose uptake and thermogenesis are needed to fully effect glucose homeostasis.

## Graphical abstract

**Figure:**
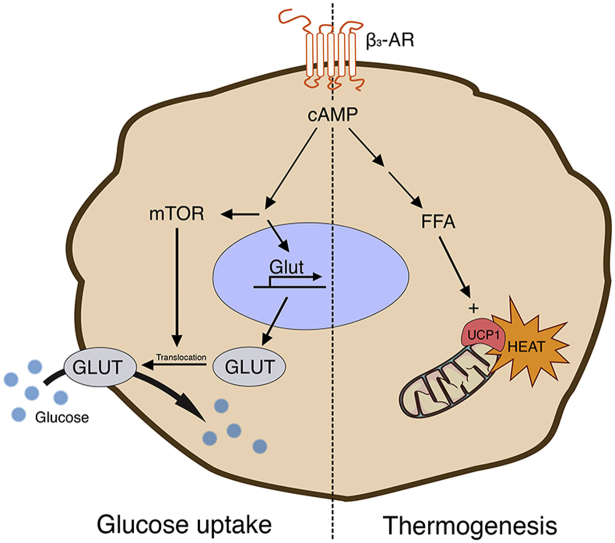

## 1 Introduction

Brown adipose tissue (BAT) can combust (surplus) energy through uncoupled respiration mediated by uncoupling protein 1 (UCP1). The possibility to use this ability to ameliorate obesity and diabetes has long been discussed [1], [2], [3]. However, as BAT was thought not to be present in adult humans, this strategy was largely disregarded. The realization that [2-^18^F]-2-fluoro-2-deoxy-d-glucose positron emission scanning tomography (PET-FDG) data, from a series of clinical investigations indicated the presence of active BAT in adult humans [4], and the subsequent confirmations of this new paradigm [5], [6], [7], [8], [9] have suggested new avenues for ameliorating obesity and diabetes, through BAT activity.

The PET-FDG technique has been applied clinically to detect cancer, based on the tenet that many cancer cells display a high metabolism, mainly fueled by glucose. Thus, the metabolism of the cells functions to clear glucose from the blood, and the accumulation of [^18^F]FDG in the cells is interpreted to indicate a high rate of metabolism. Accordingly, the uptake of [^18^F]FDG in BAT has generally been equated with thermogenic activity [4], as this activity would result in uptake of glucose, driven by the catabolic thermogenesis. In practice, no other methods for examining BAT activity, location, and amount in humans are currently routinely in use. However, the assumption that glucose uptake directly mirrors thermogenic activity [10] may not be correct.

BAT thermogenesis is activated by adrenergic stimulation [2]. Apparently, a large increase in BAT glucose uptake (as monitored by PET-FDG scans) is observed after treatment of humans with sympathomimetics [11] and in pheochromocytoma patients [12], [13], [14]. Particularly, a strong pattern of BAT glucose uptake is seen in PET scans after activation of β_3_-adrenergic receptors [15]. The aim of the present study was to investigate whether such adrenergically induced glucose uptake adequately reflects thermogenesis. We found, unexpectedly, that acute β_3_-adrenergic stimulation induces glucose uptake in BAT *independently* of the presence and activation of UCP1 and thus *independently* of thermogenesis. Our results thus demonstrate that acute glucose uptake is a mechanism separate from thermogenesis.

## 2 Results

### 2.1 UCP1 is not essential for the glucose uptake into BAT as visualized by PET-FDG

To verify the relationship between thermogenesis and β_3_-adrenergically induced glucose uptake, we used mice possessing UCP1 (wildtype), that is, mice with a capacity for nonshivering thermogenesis, or mice without (UCP1(−/−)), that is, mice without the capacity for nonshivering thermogenesis. UCP1 is essential for adrenergically induced thermogenesis in brown adipocytes [16]. Adult humans, through housing and clothing, are generally exposed only to thermoneutral conditions. Therefore, to approach human conditions in our *in vivo* rodent studies, we used wildtype and UCP1(−/−) mice adapted to thermoneutrality. Even though the demand for thermogenesis for protection against cold is eliminated in such mice, these mice still possess UCP1 in their BAT (see below). We used these mice to follow glucose uptake into BAT. To stimulate adrenergically induced glucose uptake, the β_3_-adrenergic agonist CL-316,243 was used [17]. We chose this agent instead of a physiologically induced adrenergic stimulation, such as exposure of the mice to cold, because acute cold stimulation not only activates BAT but also leads to shivering, and the shivering muscles would compete for glucose uptake. Similarly, we chose a selective β_3_-agonist, as norepinephrine itself would affect many other cell types in the body through different adrenergic receptors. In contrast, practically only adipose tissues possess β_3_-adrenergic receptors [18].

**Figure 1:**
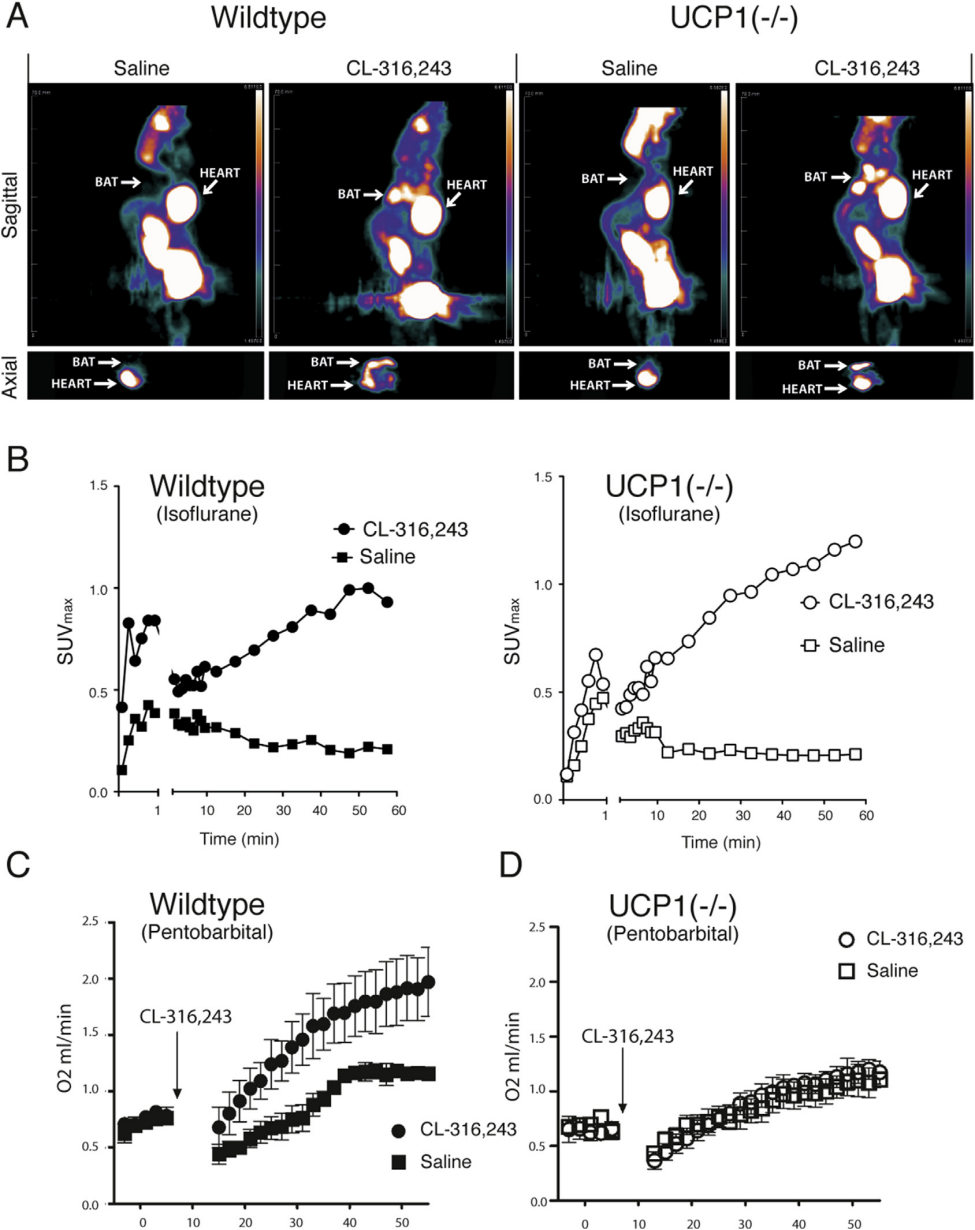
**Adrenergically induced glucose uptake in BAT is independent of thermogenic competence**. (A–B) Representative figures of wildtype and UCP1(−/−) adult lean mice that were anesthetized with isoflurane and in which the uptake of [^18^F]FDG was followed for 60 min in a MicroPET scanner. (A) Sagittal and axial PET images of the [^18^F]FDG uptake in two representative mice treated with saline (control) and CL-316,243 (1 mg/kg) on separate days. The images are sums of the last 25 min of the measurements. (B) Time activity curves of the [^18^F]FDG uptake in BAT in the mouse in A (SUVmax = maximum Standardized Uptake Values) over the entire 60 min scan. (C–D) CL-316,243-stimulated (1 mg/kg per body weight) oxygen consumption in (C) wildtype (n = 3–4) and (D) UCP1(−/−) (n = 3–4) mice housed at thermoneutrality (30 °C) for 3 weeks. The mice were anesthetized with pentobarbital (55 mg/kg i.p.). The arrow indicates i.p. injection of saline or of CL-316,243 at a dose of 1 mg per kg body weight.

In Figure 1A, the leftmost PET-FDG scan shows the last 25 min of the measurement of glucose uptake in wildtype mice anesthetized with isoflurane and treated only with saline. The scan clearly shows an uptake of glucose in the heart, with no visible uptake in the suspected BAT areas. However, when these wildtype mice were treated with the β_3_-adrenergic agonist CL-316,243 (next scan), there was a very marked uptake in the areas corresponding to BAT. The time activity curve for the BAT area (Figure 1B, left) shows how – after initial distribution during the first minutes after the i.v. injection – the uptake continuously increases during the 1 h scan in the CL-316,243-treated mice, whereas no further net uptake occurs after ≈20 min in the control mice.

As thermogenesis in BAT is β_3_-adrenergically induced [2], this result would be expected if the glucose uptake simply reflected thermogenesis. However, these mice were isoflurane-anesthetized according to laboratory routines, and isoflurane abolishes BAT-derived thermogenesis [19], [20], [21], [22]. Thus, the implication of these experiments would unexpectedly be that the observed glucose uptake is thermogenesis-independent.

The unexpected conclusion from the above experiments was verified when we performed the experiments in UCP1(−/−) mice. As thermogenesis in BAT is fully UCP1-dependent [16], any CL-316,243-induced glucose uptake in the BAT of the UCP1(−/−) mice must occur independently of thermogenesis. As seen in the two right-side scans (Figure 1A), the results in the UCP1(−/−) mice were qualitatively identical to those in wildtype mice, confirming that β_3_-adrenergically induced glucose uptake into BAT is UCP1- (and thus thermogenesis-) independent. Similarly, Figure 1B (right panel) shows the time activity curve for the [^18^F]FDG uptake in the area that corresponds to the BAT area in the UCP1(−/−) mice. Comparison of the time activity curves in Figure 1B demonstrates that the glucose uptake during the conditions used here is temporally very similar whether the tissue contains UCP1 or not. The same trend is demonstrated for all wildtype and UCP1(−/−) mice (S1).

A possible explanation for the uptake of [^18^F]FDG even in the mice not possessing UCP1 could be that the CL-316,243 stimulates some kind of UCP1-independent thermogenesis in BAT that would also utilize glucose and thus lead to glucose uptake. Indeed, there have been studies published that have been interpreted to indicate a potential for UCP1-independent thermogenesis [23], [24], [25], [26] (although not as such located to BAT). To address whether UCP1-independent thermogenesis would occur during the experiments described here, oxygen consumption in control and UCP1(−/−) mice stimulated with CL-316,243 was measured. These experiments (performed in pentobarbital-anesthetized mice in which thermogenesis is not inhibited [21]) clearly show (Figure 1C) that whereas CL-316,243 is a potent thermogenesis stimulator in wildtype mice, there is no indication of any induction of any thermogenesis by CL-316,243 in mice without UCP1. Thus, principally in agreement with the conclusion from other studies [27], [28], no alternative UCP1-independent thermogenic pathway exists that could explain the glucose uptake in BAT.

### 2.2 UCP1 is not essential for glucose uptake into BAT as examined with [^3^H]-2-deoxy-D-glucose ([^3^H]-2DG)

**Figure 2:**
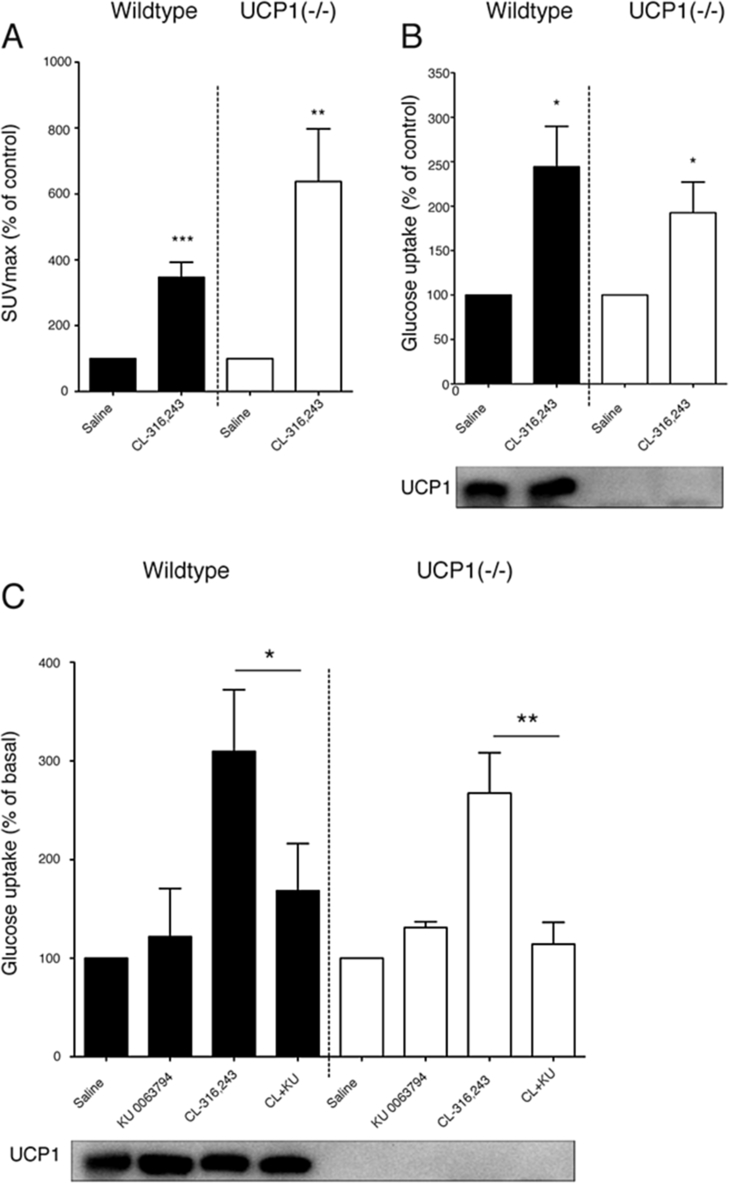
**β-Adrenergic stimulation of glucose uptake in BAT is dependent on mTOR activity.** (A) [^18^F]FDG uptake over the last 25 min of the PET scans (maximum Standardized Uptake Value, SUV_max_) expressed as % of saline-injected mice in BAT in wildtype and UCP1(−/−) mice treated with CL-316,243 (n = 5). For each mouse, the values obtained after saline injection (control) were set to 100%. Error bars represent SEM; **p < 0.01; ***p < 0.001 indicate significant effects of CL-316,243 (Student's paired *t*-test). Absolute mean of SUVmax for WT saline-control 0.42 ± 0.04 SUV/body weight and UCP1(−/−) control 0.46 ± 0.06. (B) Glucose uptake in BAT measured with [^3^H]-2DG in mice. Wildtype and UCP1(−/−) mice were treated with saline or CL-316,243 (1 mg/kg) 20 min prior to [^3^H]-2DG injection. The experiment was terminated 1 h after [^3^H]-2DG injection, and the amount of [^3^H]-2DG uptake was analyzed by liquid scintillation counting (n = 7). The mean control uptake was set to 100 in both genotypes. Absolute mean WT control values were 38104 ± 4824 CPM/g w.w. and UCP1(−/−) control 45782 ± 6601 CPM. Error bars represent SEM; *p < 0.05: significant effect of CL-316,243 (Student's unpaired *t*-test). Western blot shows the lack of UCP1 in BAT from UCP1(−/−) mice. (C) Wildtype and UCP1(−/−) mice were injected (i.p.) with KU-0063794 (10 mg/kg) or with DMSO 10 min prior to acute treatment with CL-316,243 (1 mg/kg). After 20 min, the mice were injected with [^3^H]-2DG. The experiment was terminated after 1 h, after which BAT was dissected out and the uptake was measured as in Figure 1B. Western blot below shows that UCP1 protein levels were not altered by the acute KU-0063794 and/or CL-316,243 treatment. Absolute mean values for WT control were 20934 ± 2474 CPM/g w.w. and for UCP1(−/−) control 6535 ± 695. Error bars represent SEM; *p < 0.05; **p < 0.01 for effect of KU-0063794, Student's unpaired *t*-test (n = 5). The experiment was performed in 5 cohorts of 4 animals (one for each treatment and genotype). In each experiment and genotype, the uptake in control (DMSO- plus saline-treated) mice was set to 100%.

The above PET-FDG experiments indicated that the β_3_-adrenergically induced glucose uptake is thermogenesis-independent. Quantification of a series of such experiments shows that the β-adrenergically stimulated increase in [^18^F]FDG uptake is significant in both wildtype and UCP1(−/−) mice (Figure 2A), i.e. the effect is UCP1-independent. However, it is conceivable that a significant thermogenesis-dependent glucose uptake does exist but it is not observable under the experimental conditions used above, due to the isoflurane anesthesia. We therefore attempted to measure glucose uptake by PET-FDG scans in mice anesthetized with pentobarbital (that does not inhibit thermogenesis in BAT [20]), but many of these mice did not tolerate the experimental conditions during imaging.

We therefore elected to examine glucose uptake in BAT by another method. For this, pentobarbital-anesthetized mice were injected with saline or CL-316,243 and then with [^3^H]-2DG. After 1 h, we measured the total uptake of [^3^H]-2DG in the interscapular BAT. Also here, CL-316,243 treatment increased glucose uptake in UCP1(−/−) mice to essentially the same extent as in wildtype mice (Figure 2B). Thus, even in the absence of isoflurane, the induced increase in glucose uptake into BAT was independent of UCP1.

### 2.3 The UCP1-independent pathway for adrenergically induced glucose uptake

The above experiments taken together clearly show that the acute adrenergically induced increase in glucose uptake into BAT is not due to UCP1 activity. Therefore, an alternative mechanism for regulation of glucose uptake must exist. Studies in cultured mouse brown adipocytes have shown that β-adrenergic stimulation leads to glucose uptake via synthesis of the glucose transporter GLUT1 and subsequent translocation of GLUT1 to the plasma membrane [29]. Such an adrenergically induced glucose uptake has also been shown to occur in human brown adipocytes [29]. The pathway (as detailed in [29]) regulating the translocation involves the mechanistic target of rapamycin (mTOR), more specifically mTORC2 (see also [30]).

To examine whether the β_3_-induced glucose uptake observed above in BAT could have been mediated via this mTOR pathway, we examined the effect of the mTOR inhibitor KU-0063794 [31] on CL-316,243-induced glucose uptake *in vivo* (Figure 2C, left part). In wildtype mice, the increase in glucose uptake induced by CL-316,243 was blocked by KU-0063794 (KU-0063794 in itself had no effect on glucose uptake). This implies that practically all glucose uptake into BAT is mediated via the mTOR pathway, most likely through mTORC2 [29], [30].

When we repeated these experiments in UCP1(−/−) mice, the uptake and KU-inhibition pattern was the same (Figure 2C, right part) as in the wildtype mice.

### 2.4 Even in mice made prediabetic, acute β_3_-induced glucose uptake is UCP1-independent

Since BAT has an extremely high capacity to import glucose [32], [33], [34], [35], [36], activation of glucose uptake in BAT has been suggested as a possible approach to ameliorate diseases with impaired glucose tolerance, such as type 2 diabetes. Accordingly, treatment of animals with the β_3_-adrenoceptor agonist CL-316,243 used above has demonstrated anti-diabetic effects [37], [38], [39], [40]. This may be implied to occur due to CL-316,243-induced UCP1 activation, leading to highly increased combustion of glucose in the cells, leading to a high glucose uptake. However, based on the studies above, it may be questioned whether this glucose disposal effect is dependent of thermogenesis – or simply is due to stimulated glucose uptake.

Therefore, to examine the significance of UCP1 activity for the diabetes-ameliorating effect of β_3_-agonist treatment, the effect of CL-316,243 treatment was investigated in mice made prediabetic. For this, wildtype and UCP1(−/−) mice were kept on a high-fat diet for more than six months. The wildtype and the UCP1(−/−) mice weighed approximately the same after this extensive period of high-fat feeding (wildtype, 45 ± 1 g; UCP1(−/−) mice, 43 ± 6 g). Both wildtype and UCP1(−/−) mice showed elevated fasting glucose levels at around 10 mM (see below), i.e. were pre-diabetic, compared to non-pre-diabetic mice with a fasting glucose level between 5.5 and 8.3 mM [41].

**Figure 3:**
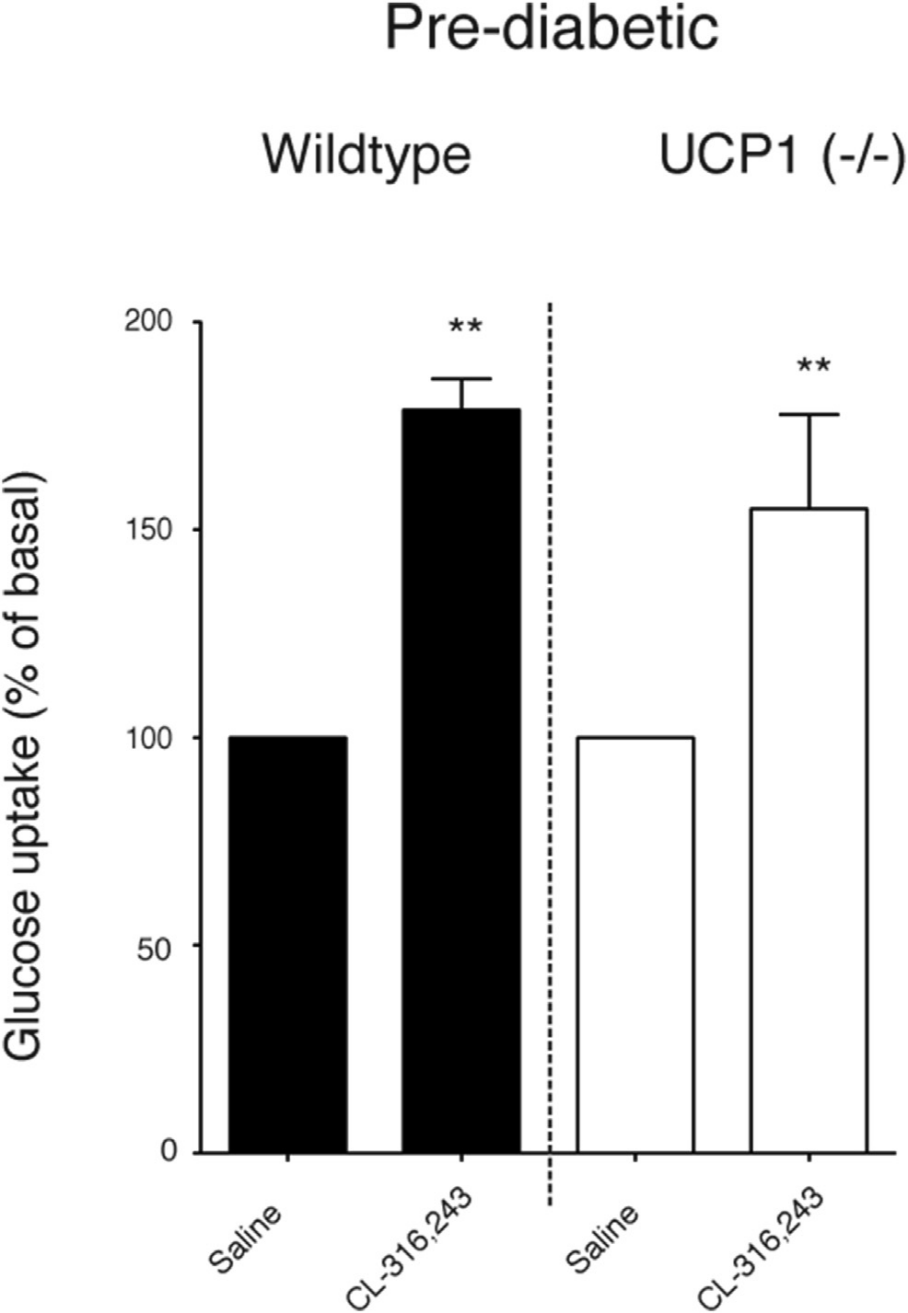
**In mice made prediabetic, acute β**_**3**_**-adrenergic agonist induces UCP1-independent glucose uptake.** Wildtype and UCP1(−/−) mice were kept on a high-fat diet for 6 months and examined for β_3_-adrenergically induced glucose uptake. The mice were injected with CL-316,243 (1 mg/kg, i.p injection) 20 min prior to injection of [^3^H]-2DG (n = 4 in each group). The mean control uptake was set to 100 in both genotypes. Absolute mean values for WT control glucose uptake were 19733 ± 1337 CPM/g w.w. and for UCP1(−/−) control 36159 ± 5524. Error bars represent SEM; **p < 0.01 (Student's unpaired *t*-test).

To examine whether the β_3_-induced glucose uptake still occurred in these mice and to examine whether this uptake was UCP1-dependent, glucose uptake measurements were performed as above with [^3^H]-2DG and CL-316,243. As seen in Figure 3, acute β_3_-treatment increased glucose uptake to the same extent in both wildtype and UCP1(−/−) BAT, even in these prediabetic mice. Thus, UCP1 was not necessary for the induced increase in glucose uptake.

### 2.5 UCP1 is essential for the ameliorating effects of prolonged β_3_-agonist treatment on glucose levels in prediabetic mice

To examine the effects of prolonged β_3_-adrenergic treatment on glucose homeostasis, the prediabetic wildtype and UCP1(−/−) mice (as above) were injected with CL-316,243 twice a day for four days. This led to a minor decrease in body weight for both genotypes (3.6 g for wildtype, 1.7 g for UCP1(−/−)).

**Figure 4:**
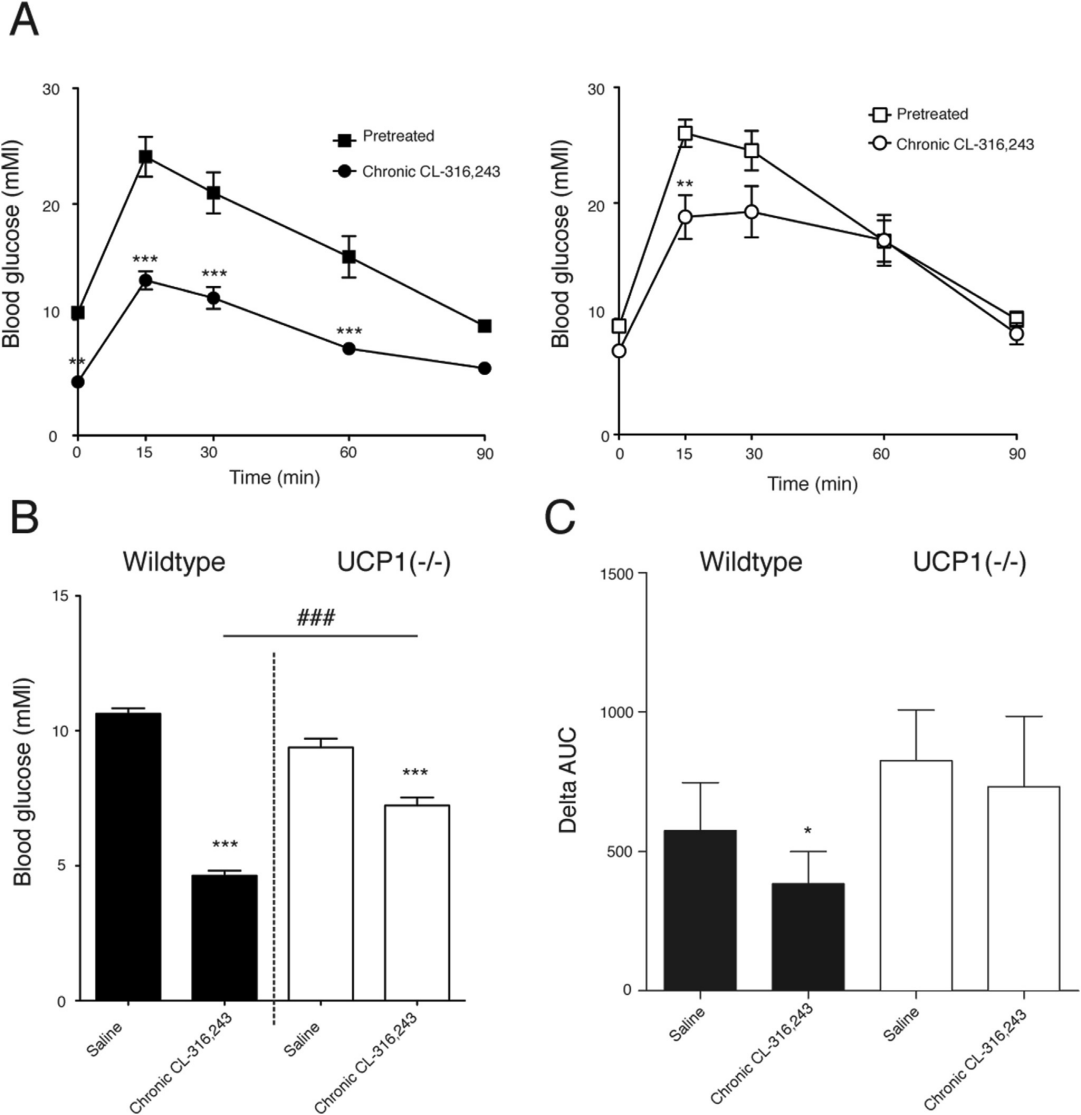
**Prolonged β**_**3**_**-adrenergic agonist treatment ameliorates blood glucose levels in prediabetic mice**. Wild-type and UCP1(−/−) mice were kept on a high-fat diet for 6 months and examined for fasting blood glucose levels and exposed to a glucose tolerance test before and after prolonged CL-316,243 treatment. (A) Glucose tolerance test in wildtype and UCP1(−/−) mice before and after the prolonged treatment with CL-316,243 (n = 6). Repeated measures ANOVA analysis yielded significant interaction between treatment and time in both wildtype and UCP1 (P < 0.05). Error bars represent SEM; *p < 0.05; **p < 0.01; ***p < 0.001 versus pretreatment (repeated measurements ANOVA). (B) Blood glucose levels in wildtype and UCP1(−/−) mice before and after prolonged treatment with CL-316,243 (1 mg/kg twice a day for four days, no CL-316,243 treatment on the experimental day (n = 6) (Student's paired *t*-test between genotypes, unpaired between genotypes #). (C) Area under the curve (AUC) (delta area above initial values) from the glucose tolerance test for wildtype and UCP1(−/−) mice before and after the prolonged treatment with CL-316,243 (n = 6). Error bars represent SEM; *p < 0.05; **p < 0.01; ***p < 0.001 versus pretreatment (Student's paired *t*-test).

During a standard glucose tolerance test (2 g glucose/kg body weight injected intraperitonally), both untreated wildtype and UCP1(−/−) mice (Figure 4A) responded with an increase in blood glucose leading to values that exceeded 25 mM. After the prolonged treatment of both wildtype and UCP1(−/−) mice with CL-316,243, the glucose values observed during the glucose tolerance test were significantly lower. This was mainly because the CL-316,243 treatment resulted in remarkably decreased fasting blood glucose levels in UCP1-expressing mice, from the diabetic 11 mM down to euglycemic levels of about 4 mM (Figure 4B). The decrease was also observed but was less pronounced in UCP1(−/−) mice: from 9 mM down to 7 mM. Analyzing the area under the curves from the GTT of both genotypes (Figure 4C) demonstrated that there was also an effect of CL-316,243 on the GTT in wildtype mice but not in the UCP1(−/−) mice. Thus, UCP1 is essential to achieve the full capacity for amelioration of fasting blood glucose levels and for enhanced glucose clearance after prolonged β_3_-treatment.

## 3 Discussion

In this study, we demonstrate that acute adrenergic stimulation of BAT *in vivo* can induce glucose uptake through the previously described mTOR pathway [29] and that the activity of this acute pathway can occur independently of the presence or function of UCP1. These data have implications for several issues: understanding mechanisms regulating glucose uptake in BAT, interpretation of activity in BAT in PET-FDG scans, and the possibility of utilizing BAT as an anti-diabetic tissue.

### 3.1 PET-FDG scans of BAT do not necessarily reflect thermogenic activity

PET imaging with [^18^F]FDG is currently the only method used for *in vivo* detection of metabolic activity of BAT and for quantifying metabolism in BAT in humans [4]. The studies presented here clearly demonstrate that acute glucose uptake cannot unreflectingly be considered to be a measure of BAT thermogenesis, to be an indicator of the localization of BAT depots or be used for quantifying BAT amounts. Most of the areas in humans early observed to become highly visible in PET-FDG scans have been demonstrated to also possess UCP1. However, our study indicates that there nevertheless may not be a proportionality between glucose uptake and thermogenesis, and a parallelism between increased [^18^F]FDG uptake and brown-fat thermogenesis should not necessarily be expected. Thus, some reports indicating increased BAT after e.g. β_3_-treatment [11], [15] may not necessarily reflect such an increase but merely an increased glucose uptake.

### 3.2 UCP1-dependent and UCP1-independent adrenergic effects in BAT

The present studies are paralleled by others pointing to the risk of considering events in BAT secondary to thermogenesis/UCP1 activity. Thus, blood flow through the tissue cannot be read out as a simple thermogenesis indicator [42], and even VEGF gene expression [43], [44] and the vascularization of the tissue [45] are not caused by UCP1 activity and, thereof, lowered oxygen pressure. Activation of the sympathetic nervous system stimulates parallel processes, such as thermogenesis and, for instance, glucose uptake, which do therefore not necessarily need to be functionally connected. In all these cases, the reason is that the processes followed are regulated via the sympathetic nervous system, independently of the activation of thermogenesis as such. Thus, generally, measures of glucose uptake (and e.g. blood flow) will coincide with thermogenesis, but neither increased glucose uptake nor blood flow needs to be a consequence of thermogenesis. Similarly, the localization of BAT may not fully follow that of adrenergically (or physiologically) induced glucose uptake, which, at least theoretically, could occur in adipocytes lacking (or having only low amounts of) UCP1 and mitochondria.

### 3.3 Regulation of glucose uptake in BAT

The present study demonstrates that glucose uptake, at least acutely, is an independently regulated, sympathetically driven process. In this respect, the present studies are also an *in vivo* extension of earlier studies showing that UCP1 is not a prerequisite for adrenergically induced glucose uptake in cultured brown adipocytes [46] or in intact mice [47]. In contrast, Inokuma et al. [48] showed indications that in UCP1(−/−) mice, norepinephrine lost its ability to enhance glucose uptake rate into BAT, but this was mainly due to an unexpected increase in the basal uptake rate. Jeanguillaume et al. [49] observed intriguing differences between the effect of the absence of UCP1 on glucose uptake in male and female mice; however, we have not observed such a difference (data not shown). Therefore, our results provide evidence that acute, adrenergically-induced glucose uptake and thermogenesis are two separately regulated processes in BAT and that the induced acute glucose uptake can occur via mTOR mediation, independently of thermogenic activity in the brown-fat cells.

### 3.4 Utilizing BAT as an anti-diabetic tissue

The present studies point clearly to a possibility of utilizing glucose uptake into BAT to ameliorate diabetes. Notably, we found that even in mice made prediabetic, a few days' treatment with a β_3_-agonist led to near normalization of fasting glucose levels and a significant decrease in AUC of the GTTs for the wildtype mice, principally in accordance with earlier results [37], [38], [39]. We additionally show here that this effect is only fully active in mice that possess UCP1. In mice without UCP1, the acute glucose uptake is still present but the capacity of the cells to maintain the glucose uptake will possibly deteriorate due to the large amount of excess energy the cells have to store, likely in the form of glycogen [50]. In brown-fat cells with UCP1, this storage limitation will not be relevant, as the glucose will be oxidized [51]. Thus, it is evident that both glucose uptake and thermogenesis are needed to fully utilize the capacity of the cell to influence glucose homeostasis and that both should be stimulated to reach maximum glucose uptake capacity. Only then can BAT fully be exploited [52] in fighting the metabolic syndrome.

## 4 Experimental procedures

### 4.1 Animals

UCP1(−/−) mice (on a C57Bl/6 background for more than 10 generations), originally derived from those described by Enerbäck et al. [53], and UCP1-wildtype mice (C57Bl/6), were bred at the Stockholm University Experimental Core Facility. Wildtype and UCP1(−/−) mice were kept as separated lines with regular backcrosses. Mice (4–7 months old) of both genders were housed at 30 °C for at least 3 weeks prior to the experiments, with a 12:12-h light–dark cycle, and with free access to food and water during the whole study. The mice were single-caged 1 day prior to the experiment.

For the study of the effect in mice made prediabetic, mice were fed a high-fat diet (45% kcal energy by fat, Research Diets D12451, New Jersey, USA) for at least 6 months at 30 °C.

All studies were approved by the Animal Ethics Committee of the North Stockholm Region.

### 4.2 MicroPET studies

UCP1(−/−) mice and UCP1-wildtype mice (C57Bl/6) as above were fasted for 5 h prior to microPET imaging (Focus 120, CTI Concorde Microsystems Inc.). The mice were weighed and anesthetized with isoflurane (5% initially and then 1.5% to maintain anesthesia, mixed with 6:4 air/O_2_). Then the blood glucose levels were measured. The mice were placed prone on a heating pad (37 °C) on the microPET camera bed, with most of the body in the field-of-view. Either CL-316,243 (1 mg/kg) or saline (on the same mice on another occasion) was injected intraperitoneally 20 min prior to [^18^F]FDG (obtained from daily clinical production at Karolinska University Hospital, 7–8 MBq per mouse, maximum volume 200 μl), which was administered by bolus injection via the tail vein. Emission data were collected for 60 min in list mode. Data were processed using MicroPET Manager (CTI Concorde Microsystems). PET data were acquired in fully three-dimensional (3D) mode, and images were reconstructed by standard 2D filtered back projection using a ramp filter. Data were corrected for randoms, dead time and decay. BAT regions of interest (ROIs) were drawn manually on the images. First a ROI was drawn in the activated BAT location and then the same volume was drawn on the saline treatment image and images were compared to acquire the same approximate position. The ROI counts were normalized to the injected dose and mouse body weight and calculated as mean, maximum and minimum standard uptake values (SUV) using Inveon Research Workplace software (Siemens Medical Solutions). SUVmax values were used to reduce influences of spill-in and spill-over effects from adjacent tissues on the quantifications.

### 4.3 Metabolic studies

Mice housed at housed at thermoneutrality (30 °C) for 3 weeks were anaesthetized with pentobarbital (55 mg/kg i.p.) and were transferred to metabolic chambers as previously described [54]. The mice were measured at 33 °C to obtain basal values during 10 min. After these basal readings, the mice were removed from the metabolic chambers for a short time (6 min) and injected intraperitoneally with CL-316,243 (1 mg/kg) or saline before being returned to the metabolic chambers for 60 min.

### 4.4 [^3^H]-2DG uptake in mice

For measurement of glucose uptake in mice with and without UCP1, the mice were fasted for 5 h at 30 °C prior to the study. All mice were anesthetized with an intraperitoneal (i.p.) injection of pentobarbital (55 mg/kg body weight). Where indicated, mice were injected i.p. with KU-0063794 (10 mg/kg) or with DMSO 10 min before injection with CL-316,243 (1 mg/kg body weight, Sigma Aldrich) or saline. Twenty min later, [^3^H]-2DG (130 μCi/kg body weight) was injected. Mice were sacrificed after 1 h, interscapular BAT was dissected out, weighed, and a piece of the tissue digested with 0.5 M NaOH overnight. 10% of the cell lysate was mixed with scintillation cocktail (Emulsifier-Safe, Perkin Elmer), and the amount of [^3^H]-2DG in the tissues was detected with a liquid scintillation analyzer (Tri-Carb 2800 TR, Perkin Elmer, Waltham, MA, USA) with a scintillation measuring time of 3 min. CPM values obtained were in the range 70–4000.

### 4.5 Prolonged treatment with CL-316,243

Groups of wildtype and UCP1(−/−) mice, fed a high fat diet (at least 6 months), were weighed and injected i.p. with 1 mg/kg body weight CL-316,243 twice a day, for 4 days in a 30 °C room, or with saline. On day 0 and 5, the mice were starved for 5 h at 30 °C and weighed before a glucose tolerance test was performed (2 g glucose i.p. per kg body weight, at 30 °C). Blood from the tail vein was drawn to measure blood glucose values with a glucometer (AccuCheck, Biochemical systems international Srl., Florence, Italy).

### 4.6 Immunoblotting

BAT was homogenized as previously described [54]. Immunoblotting was performed as previously described [55]. The primary UCP1 antibody was produced in rabbit against C-terminal decapeptide, diluted 1:5000. The primary antibody was detected with a secondary antibody (horseradish peroxidase-linked anti-rabbit IgG, Cell Signaling) diluted 1:2000.

### 4.7 Statistics

All results are expressed as means ± SEM. The statistical significance of differences between groups was analyzed by Student's two-tailed *t*-test or with repeated measures ANOVA, as indicated.

## Figures and Tables

**Supplementary Figure 1:**
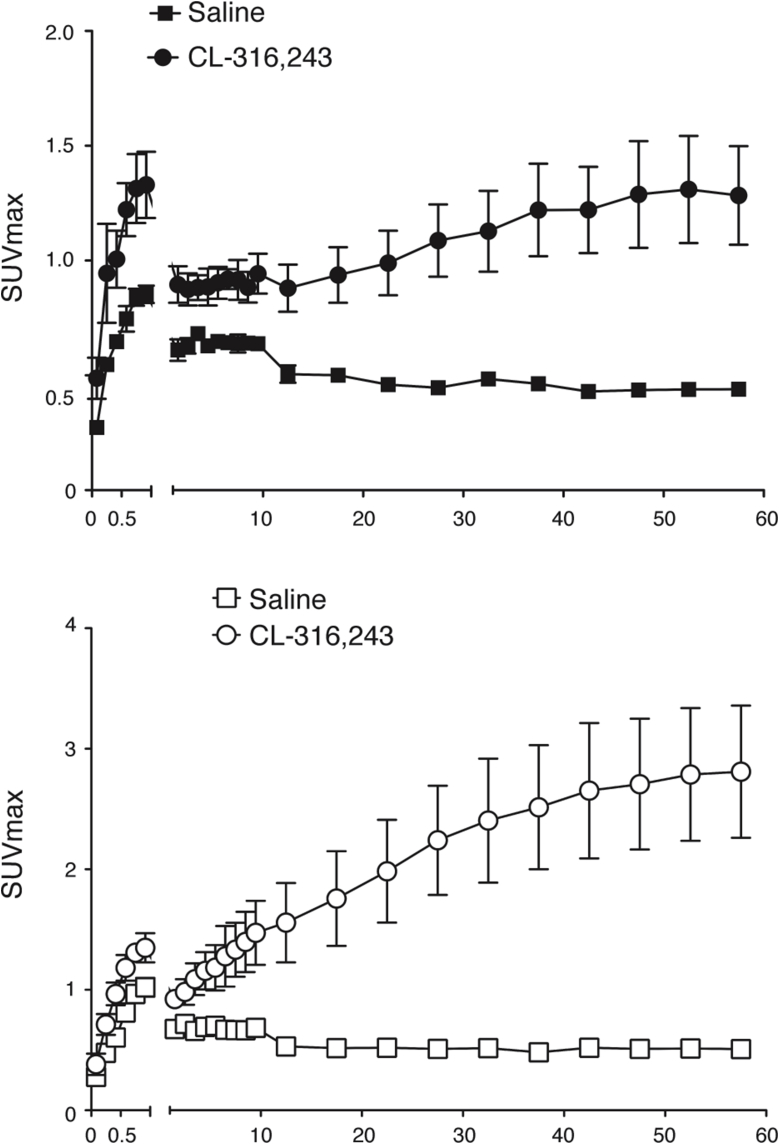
Representative figures of wildtype and UCP1(−/−) adult lean mice, that were anesthetized with isoflurane and in which the uptake of [^18^F]FDG was followed for 60 min in a MicroPET scanner. (A) Sagittal and axial PET images of the [^18^F]FDG uptake in two representative mice treated with saline (control) and CL-316,243 (1 mg/kg) on separate days. The images are sums of the last 25 min of the measurements. (B) Time activity curves of the [^18^F]FDG uptake in BAT in the mouse in A (SUVmax = maximum Standardized Uptake Values) over the entire 60 min scan.

## References

[1] Himms-Hagen J. (1984). Thermogenesis in brown adipose tissue as an energy buffer. Implications for obesity. The New England Journal of Medicine.

[2] Cannon B., Nedergaard J. (2004). Brown adipose tissue: function and physiological significance. Physiological Reviews.

[3] Yen T.T. (1994). Antiobesity and antidiabetic beta-agonists: lessons learned and questions to be answered. Obesity Research.

[4] Nedergaard J., Bengtsson T., Cannon B. (2007). Unexpected evidence for active brown adipose tissue in adult humans. American Journal of Physiology Endocrinology and Metabolism.

[5] Cypess A.M., Lehman S., Williams G., Tal I., Rodman D., Goldfine A.B. (2009). Identification and importance of brown adipose tissue in adult humans. The New England Journal of Medicine.

[6] Saito M., Okamatsu-Ogura Y., Matsushita M., Watanabe K., Yoneshiro T., Nio-Kobayashi J. (2009). High incidence of metabolically active brown adipose tissue in healthy adult humans: effects of cold exposure and adiposity. Diabetes.

[7] van Marken Lichtenbelt W.D., Vanhommerig J.W., Smulders N.M., Drossaerts J.M., Kemerink G.J., Bouvy N.D. (2009). Cold-activated brown adipose tissue in healthy men. The New England Journal of Medicine.

[8] Virtanen K.A., Lidell M.E., Orava J., Heglind M., Westergren R., Niemi T. (2009). Functional brown adipose tissue in healthy adults. The New England Journal of Medicine.

[9] Zingaretti M.C., Crosta F., Vitali A., Guerrieri M., Frontini A., Cannon B. (2009). The presence of UCP1 demonstrates that metabolically active adipose tissue in the neck of adult humans truly represents brown adipose tissue. FASEB Journal.

[10] Marette A., Bukowiecki L.J. (1991). Noradrenaline stimulates glucose transport in rat brown adipocytes by activating thermogenesis. Evidence that fatty acid activation of mitochondrial respiration enhances glucose transport. The Biochemical Journal.

[11] Carey A.L., Formosa M.F., Van Every B., Bertovic D., Eikelis N., Lambert G.W. (2013). Ephedrine activates brown adipose tissue in lean but not obese humans. Diabetologia.

[12] Taieb D., Sebag F., Barlier A., Tessonnier L., Palazzo F.F., Morange I. (2009). 18F-FDG avidity of pheochromocytomas and paragangliomas: a new molecular imaging signature?. Journal of nuclear medicine : official publication. Society of Nuclear Medicine.

[13] Ramacciotti C., Schneegans O., Lang H., Lindner V., Claria M., Moreau F. (2006). Diffuse uptake of brown fat on computed-tomography coupled positron emission tomoscintigraphy (PET-CT) for the exploration of extra-adrenal pheochromocytoma. Annales d'Endocrinologie.

[14] Fukuchi K., Tatsumi M., Ishida Y., Oku N., Hatazawa J., Wahl R.L. (2004). Radionuclide imaging metabolic activity of brown adipose tissue in a patient with pheochromocytoma. Experimental and Clinical Endocrinology & Diabetes.

[15] Cypess A.M., Weiner L.S., Roberts-Toler C., Franquet Elia E., Kessler S.H., Kahn P.A. (2015). Activation of human brown adipose tissue by a beta3-adrenergic receptor agonist. Cell Metabolism.

[16] Matthias A., Ohlson K.B., Fredriksson J.M., Jacobsson A., Nedergaard J., Cannon B. (2000). Thermogenic responses in brown fat cells are fully UCP1-dependent. UCP2 or UCP3 do not substitute for UCP1 in adrenergically or fatty scid-induced thermogenesis. The Journal of Biological Chemistry.

[17] Himms-Hagen J., Cui J., Danforth E., Taatjes D.J., Lang S.S., Waters B.L. (1994). Effect of CL-316,243, a thermogenic beta 3-agonist, on energy balance and brown and white adipose tissues in rats. The American Journal of Physiology.

[18] Krief S., Lonnqvist F., Raimbault S., Baude B., Van Spronsen A., Arner P. (1993). Tissue distribution of beta 3-adrenergic receptor mRNA in man. The Journal of Clinical Investigation.

[19] Ohlson K.B., Mohell N., Cannon B., Lindahl S.G., Nedergaard J. (1994). Thermogenesis in brown adipocytes is inhibited by volatile anesthetic agents. A factor contributing to hypothermia in infants?. Anesthesiology.

[20] Ohlson K.B., Lindahl S.G., Cannon B., Nedergaard J. (2003). Thermogenesis inhibition in brown adipocytes is a specific property of volatile anesthetics. Anesthesiology.

[21] Ohlson K.B., Shabalina I.G., Lennstrom K., Backlund E.C., Mohell N., Bronnikov G.E. (2004). Inhibitory effects of halothane on the thermogenic pathway in brown adipocytes: localization to adenylyl cyclase and mitochondrial fatty acid oxidation. Biochemical Pharmacology.

[22] Dicker A., Ohlson K.B., Johnson L., Cannon B., Lindahl S.G., Nedergaard J. (1995). Halothane selectively inhibits nonshivering thermogenesis. Possible implications for thermoregulation during anesthesia of infants. Anesthesiology.

[23] Ukropec J., Anunciado R.P., Ravussin Y., Hulver M.W., Kozak L.P. (2006). UCP1-independent thermogenesis in white adipose tissue of cold-acclimated Ucp1−/− mice. The Journal of Biological Chemistry.

[24] Keipert S., Jastroch M. (2014). Brite/beige fat and UCP1-is it thermogenesis?. Biochimica et Biophysica Acta.

[25] Bal N.C., Maurya S.K., Sopariwala D.H., Sahoo S.K., Gupta S.C., Shaikh S.A. (2012). Sarcolipin is a newly identified regulator of muscle-based thermogenesis in mammals. Nature Medicine.

[26] Granneman J.G., Burnazi M., Zhu Z., Schwamb L.A. (2003). White adipose tissue contributes to UCP1-independent thermogenesis. American Journal of Physiology Endocrinology and Metabolism.

[27] Golozoubova V., Hohtola E., Matthias A., Jacobsson A., Cannon B., Nedergaard J. (2001). Only UCP1 can mediate adaptive nonshivering thermogenesis in the cold. FASEB Journal.

[28] Golozoubova V., Cannon B., Nedergaard J. (2006). UCP1 is essential for adaptive adrenergic nonshivering thermogenesis. American Journal of Physiology Endocrinology and Metabolism.

[29] Olsen J.M., Sato M., Dallner O.S., Sandstrom A.L., Pisani D.F., Chambard J.C. (2014). Glucose uptake in brown fat cells is dependent on mTOR complex 2-promoted GLUT1 translocation. The Journal of Cell Biology.

[30] Albert V., Svensson K., Shimobayashi M., Colombi M., Munoz S., Jimenez V. (2016). mTORC2 sustains thermogenesis via Akt-induced glucose uptake and glycolysis in brown adipose tissue. EMBO Molecular Medicine.

[31] Garcia-Martinez J.M., Moran J., Clarke R.G., Gray A., Cosulich S.C., Chresta C.M. (2009). Ku-0063794 is a specific inhibitor of the mammalian target of rapamycin (mTOR). The Biochemical Journal.

[32] Greco-Perotto R., Zaninetti D., Assimacopoulos-Jeannet F., Bobbioni E., Jeanrenaud B. (1987). Stimulatory effect of cold adaptation on glucose utilization by brown adipose tissue. Relationship with changes in the glucose transporter system. The Journal of Biological Chemistry.

[33] Vallerand A.L., Perusse F., Bukowiecki L.J. (1990). Stimulatory effects of cold exposure and cold acclimation on glucose uptake in rat peripheral tissues. The American Journal of Physiology.

[34] Liu X., Perusse F., Bukowiecki L.J. (1994). Chronic norepinephrine infusion stimulates glucose uptake in white and brown adipose tissues. The American Journal of Physiology.

[35] Cooney G.J., Caterson I.D., Newsholme E.A. (1985). The effect of insulin and noradrenaline on the uptake of 2-[1-14C]deoxyglucose in vivo by brown adipose tissue and other glucose-utilising tissues of the mouse. FEBS Letters.

[36] Cawthorne M.A. (1989). Does brown adipose tissue have a role to play in glucose homeostasis?. The Proceedings of the Nutrition Society.

[37] de Souza C.J., Hirshman M.F., Horton E.S. (1997). CL-316,243, a beta3-specific adrenoceptor agonist, enhances insulin-stimulated glucose disposal in nonobese rats. Diabetes.

[38] Ghorbani M., Shafiee Ardestani M., Gigloo S.H., Cohan R.A., Inanlou D.N., Ghorbani P. (2012). Anti diabetic effect of CL 316,243 (a beta3-adrenergic agonist) by down regulation of tumour necrosis factor (TNF-alpha) expression. PLoS One.

[39] Yoshida T., Sakane N., Wakabayashi Y., Umekawa T., Kondo M. (1994). Anti-obesity effect of CL 316,243, a highly specific beta 3-adrenoceptor agonist, in mice with monosodium-L-glutamate-induced obesity. European Journal of Endocrinology.

[40] Wu C., Cheng W., Sun Y., Dang Y., Gong F., Zhu H. (2014). Activating brown adipose tissue for weight loss and lowering of blood glucose levels: a microPET study using obese and diabetic model mice. PLoS One.

[41] Klueh U., Liu Z., Cho B., Ouyang T., Feldman B., Henning T.P. (2006). Continuous glucose monitoring in normal mice and mice with prediabetes and diabetes. Diabetes Technology & Therapeutics.

[42] Abreu-Vieira G., Hagberg C.E., Spalding K.L., Cannon B., Nedergaard J. (2015). Adrenergically stimulated blood flow in brown adipose tissue is not dependent on thermogenesis. American Journal of Physiology Endocrinology and Metabolism.

[43] Fredriksson J.M., Lindquist J.M., Bronnikov G.E., Nedergaard J. (2000). Norepinephrine induces vascular endothelial growth factor gene expression in brown adipocytes through a beta -adrenoreceptor/cAMP/protein kinase A pathway involving Src but independently of Erk1/2. The Journal of Biological Chemistry.

[44] Fredriksson J.M., Nikami H., Nedergaard J. (2005). Cold-induced expression of the VEGF gene in brown adipose tissue is independent of thermogenic oxygen consumption. FEBS Letters.

[45] Xue Y., Petrovic N., Cao R., Larsson O., Lim S., Chen S. (2009). Hypoxia-independent angiogenesis in adipose tissues during cold acclimation. Cell Metabolism.

[46] Hutchinson D.S., Chernogubova E., Dallner O.S., Cannon B., Bengtsson T. (2005). Beta-adrenoceptors, but not alpha-adrenoceptors, stimulate AMP-activated protein kinase in brown adipocytes independently of uncoupling protein-1. Diabetologia.

[47] Hankir M.K., Kranz M., Keipert S., Weiner J., Andreasen S.G., Kern M. (2017). Dissociation between brown adipose tissue 18F-FDG uptake and thermogenesis in uncoupling protein 1 deficient mice. Journal of Nuclear Medicine.

[48] Inokuma K., Ogura-Okamatsu Y., Toda C., Kimura K., Yamashita H., Saito M. (2005). Uncoupling protein 1 is necessary for norepinephrine-induced glucose utilization in brown adipose tissue. Diabetes.

[49] Jeanguillaume C., Metrard G., Ricquier D., Legras P., Bouchet F., Lacoeuille F. (2013). Visualization of activated BAT in mice, with FDG-PET and its relation to UCP1. Advances in Molecular Imaging.

[50] Labbe S.M., Caron A., Bakan I., Laplante M., Carpentier A.C., Lecomte R. (2015). In vivo measurement of energy substrate contribution to cold-induced brown adipose tissue thermogenesis. FASEB Journal.

[51] Chondronikola M., Volpi E., Borsheim E., Porter C., Annamalai P., Enerback S. (2014). Brown adipose tissue improves whole-body glucose homeostasis and insulin sensitivity in humans. Diabetes.

[52] Nedergaard J., Bengtsson T., Cannon B. (2011). New powers of brown fat: fighting the metabolic syndrome. Cell Metabolism.

[53] Enerback S., Jacobsson A., Simpson E.M., Guerra C., Yamashita H., Harper M.E. (1997). Mice lacking mitochondrial uncoupling protein are cold-sensitive but not obese. Nature.

[54] Abreu-Vieira G., Fischer A.W., Mattsson C., de Jong J.M., Shabalina I.G., Ryden M. (2015). Cidea improves the metabolic profile through expansion of adipose tissue. Nature Communications.

[55] Lindquist J.M., Fredriksson J.M., Rehnmark S., Cannon B., Nedergaard J. (2000). Beta 3- and alpha1-adrenergic Erk1/2 activation is Src- but not Gi-mediated in Brown adipocytes. The Journal of Biological Chemistry.

