# Supplementary material for: β3-Adrenergically induced glucose uptake in brown adipose tissue is independent of UCP1 presence or activity: Mediation through the mTOR pathway
Source: Mol Metab. 2017 Mar 30;6(6):611–9. doi: 10.1016/j.molmet.2017.02.006 (PMC5444022; doi:10.1016/j.molmet.2017.02.006)
Supplement: Supplementary file 1 [file mmc1.docx]

|  |  |  | BAT weight (g) | CMP/g | CPM/tissue |
| --- | --- | --- | --- | --- | --- |
| Pre-diabetic | WT | Saline | 0.1440 ± 0.0099 | 19733 ± 1337 | 2813 ± 137 |
|  | WT | CL-316,243 | 0.1188 ± 0.0123 | 35277 ± 2745 | 4163 ± 524 |
|  | UCP1(-/-) | Saline | 0.0557 ± 0.0123 | 36159 ± 5524 | 1815 ± 152 |
|  | UCP1(-/-) | CL-316,243 | 0.0828 ± 0.0087 | 52454 ± 1496 | 4330 ± 425 |
|  |  |  |  |  |  |
| Healthy | WT | Saline | 0.1104 ± 0.0257 | 38104 ± 4824 | 4282 ± 1141 |
|  | WT | CL-316,243 | 0.1366 ± 0.0354 | 94870 ± 21657 | 13564 ± 5421 |
|  | UCP1(-/-) | Saline | 0.0432 ± 0.0063 | 45782 ± 6601 | 1964 ± 461 |
|  | UCP1(-/-) | CL-316,243 | 0.0467 ± 0.0088 | 85988 ± 19138 | 3830 ± 944 |

**Supplementary table 1.** Absolute mean values of BAT weight (g), CPM/g and CPM/tissue for healthy (Chow feed) and pre-diabetic (HF diet feed) mice. Note that tissues were not quantitatively dissected.
